# Longitudinal and radial microgradients in porosity and canal diameter in femur bone and its implications for bone regeneration and bone repair implants

**DOI:** 10.3389/fbioe.2026.1789149

**Published:** 2026-03-18

**Authors:** Xiao Zhao, Xiaojun Yu, Swera Naz, Agila Zhussupova, Dilhan M. Kalyon, Cevat Erisken

**Affiliations:** 1 Department of Chemical Engineering and Materials Science, Stevens Institute of Technology, Charles V. Schaefer, Jr. School of Engineering & Science, Hoboken, NJ, United States; 2 Department of Biomedical Engineering, Stevens Institute of Technology, Charles V. Schaefer, Jr. School of Engineering & Science, Hoboken, NJ, United States; 3 Department of Chemical and Materials Engineering, School of Engineering and Digital Sciences, Nazarbayev University, Astana, Kazakhstan

**Keywords:** bone microstructure, bone regeneration, canal diameter, cortical porosity, femur, gradient biomaterials, implant design, mechanical properties

## Abstract

**Introduction:**

Bone exhibits hierarchical structural gradients that optimize mechanical performance and regenerative potential. Longitudinal and radial variations in porosity and canal architecture of the femur influence load distribution, vascularization, and remodeling. Understanding these gradients is essential for designing scaffolds and implants that mimic native bone structure and function. This study quantified longitudinal and radial microgradients in porosity and canal diameter along the rabbit femur and explored their implications for bone regeneration and repair implant design. Rabbit femora were divided into proximal, mid-shaft, and distal regions.

**Methods:**

High-resolution micro-computed tomography quantified cortical thickness, porosity, and canal diameter along radial and longitudinal axes in micron-scale resolutions.

**Results and Discussion:**

Compressive mechanical testing of slices determined local moduli, which were correlated with microstructural parameters to establish structure–function relationships. Cortical thickness peaked at the mid-shaft and decreased toward both ends. Porosity and canal diameter increased radially toward the medullary cavity and longitudinally toward the bone ends. Upto 500 μm bone thickness from the outer surface toward modullary cavity, porosity and canal diameter ranged, respectively, from ∼5% to 40 μm at the mid-shaft to ∼40% and 110 μm at the ends. At 750 μm thickness, porosity and canal diameter ranged, respectively, from ∼5% to 50 ∼m at the mid-shaft to ∼80% and 200 μm at the ends. As expected, compressive moduli declined with increasing porosity and canal size. The mid-shaft, with the lowest porosity and smallest canals, exhibited the highest modulus of around 15 MPa, which decreased to 5 MPa toward the ends. The rabbit femur displays distinct longitudinal and radial microgradients in porosity and canal architecture that govern local stiffness. These gradients define structural benchmarks for designing functionally graded tissue engineering scaffolds and bone implants that replicate native tissue structure and stiffness transitions to promote osteoconduction, osteoinduction, osteogenesis in bone regeneration and improve osseointegration of bone implants.

## 1 Introduction

Bone quality encompasses more than just bone mineral density (BMD), incorporating critical microstructural features that determine mechanical competence and fracture resistance (Augat and Schorlemmer, 2006; Rizzoli, 2010). These include cortical thickness, porosity, and trabecular architecture (Brandi, 2009; Rachidi et al., 2008). While BMD remains a clinical standard, it often fails to predict fracture risk accurately, highlighting the importance of assessing bone microstructure (Jindal, 2018; Martin and Correa, 2010; Boskey and Imbert, 2017). Bone quality changes are associated with conditions like osteoporosis and aging, contributing to increased fracture risk (Boskey and Imbert, 2017). Advanced imaging techniques, such as high-resolution CT and MRI, can provide detailed information about bone microarchitecture, potentially improving fracture risk prediction and treatment efficacy assessment (Rizzoli, 2010; Lorentzon, 2020). Understanding and quantifying bone microstructure is vital for enhancing diagnostic accuracy, developing therapeutic interventions, and informing biomimetic strategies in implant and scaffold design (Brandi, 2009; Martin and Correa, 2010).

Cortical bone exhibits a complex hierarchical structure from the macroscale to the nanoscale, which underlies its mechanical properties and ability to resist fracture (Zimmermann and Ritchie, 2015; Fratzl et al., 2004). This hierarchical organization spans from the diaphyseal shaft to microscopic osteons and vascular canals, down to the collagen-mineral matrix (Martin, 1991; Augat and Schorlemmer, 2006). Cortical bone accounts for approximately 80% of skeletal mass and plays a critical role in load-bearing, with its mechanical performance governed by mineral content and microstructural organization (Brandi, 2009; Milovanovic et al., 2014). Microstructural parameters such as porosity, canal diameter, and cortical thickness serve as essential indicators of bone health and mechanical resilience (Schmidutz et al., 2021). The spatial distribution and density of vascular canals act as stress concentrators, influencing bone’s fatigue life (Loundagin et al., 2021). Quantitative assessment of cortical microarchitecture provides valuable insight into bone quality and structural integrity, especially in regions subject to high mechanical loads.

Recent studies have demonstrated that bone microarchitecture significantly influences its mechanical properties. Increased cortical porosity and enlarged vascular canals are associated with reduced bone strength (Augat and Schorlemmer, 2006; Schneider et al., 2013). The intracortical canal network acts as a stress concentrator, affecting fracture risk (Uniyal et al., 2021). Accordingly, bone mineral density alone is insufficient to predict fracture risk, as microarchitectural parameters improve the prediction of mechanical behavior (Wegrzyn et al., 2010).

Heterogeneity in material properties and microstructure contributes to fracture resistance and energy dissipation in bone tissue (Lloyd et al., 2015; Feng and Todoh, 2021), while age-related increases in cortical porosity and Haversian canal size negatively impact bone’s ability to withstand mechanical loads (Dong and Guo, 2001). Although porosity remains the primary determinant of cortical bone elasticity, other microarchitectural features also contribute at varying degrees (Cai et al., 2019). These findings highlight the importance of mapping compressive modulus alongside microstructural gradients, a more accurate and functional understanding of structure-function relationship can be achieved, enhancing the predictive value of microarchitectural assessment.

Spatial gradients in cortical thickness, porosity, and canal diameter are not merely an anatomical curiosity; they determine how host bone interacts with load-bearing implants such as femoral stems, tibial trays, and acetabular cups. Uniform titanium stems frequently over-stiffen the mid-shaft and under-support the more compliant metaphysis, promoting stress shielding and aseptic loosening. Implants with graded porosity, such as distally increasing axial gradients or inwardly increasing radial gradients, can improve osseointegration and reduce bone resorption (Wang et al., 2020). Designing implants with stiffness similar to cortical bone can minimize stress shielding and promote more physiological load transfer (Krishna et al., 2007; Sumner et al., 1998). Likewise, tissue-engineered scaffolds mimicking the natural bone structure, with a radial gradient of increasing porosity towards the center, have shown enhanced osteogenesis and vascularization. Studies demonstrate that higher porosity and larger pore canal diameters (>300 μm) promote bone ingrowth and vascularization *in vivo* (Karageorgiou and Kaplan, 2005; Abbasi et al., 2020). Biomimetic gradient scaffolds with fine internal pillars (∼400 μm) and coarse external pillars (∼800 μm) promote osteogenic differentiation and new bone formation (Li et al., 2024). Quantitative mapping of these gradients in the present study therefore provides design targets for next-generation joint-replacement devices and bone/osteochondral grafts.

The choice of the rabbit femur as an experimental model in this study is supported by its well-documented translational relevance to human clinical applications. Rabbits are among the most frequently utilized species in musculoskeletal research, accounting for approximately 35% of all animal studies in the field (Li et al., 2015). This preference is due to the presence of secondary Haversian systems in mature rabbit bone, a characteristic shared with humans but absent in smaller rodents (Wancket, 2015). Furthermore, the rabbit femur provides a suitable macro-environment for testing internal fixation devices and implants, as its healing patterns and mineral density profiles correlate significantly with human long bones (Pearce et al., 2007). While the accelerated bone turnover rate in rabbits, reaching skeletal maturity by 20–30 weeks, necessitates caution in direct temporal extrapolation, it serves as an excellent high-throughput screening tool for the early-phase validation of novel biomaterials and osseointegration (Scarano et al., 2024). Thus, the findings from this model provide a critical physiological and mechanical baseline for the potential development of human orthopedic implants.

The rabbit femur is widely used as a preclinical model for studying bone structure and mechanics due to its similarities to human long bones (Harrison et al., 2020; Cvetkovic et al., 2013). While not identical in scale or remodeling rates, the rabbit femur exhibits comparable cortical organization and loading environments to humans (Pearce et al., 1991; Bagi et al., 2011). The femoral shaft is particularly relevant for investigating microstructural features and biomechanics, with applications in orthopedic implant research (Huiskes, 1982; Huiskes et al., 1983). Rabbit models offer practical advantages including manageable bone size and established experimental protocols (Schindeler et al., 2018).

However, it is important to note that no animal model perfectly replicates human bone, and understanding interspecies differences is crucial for accurate extrapolation of results (Cvetkovic et al., 2013; Gauthier et al., 2018). Despite some limitations, the rabbit femur remains a valuable platform for evaluating bone microstructure and biomechanics with translational relevance to human orthopedic applications.

Recent advancements in imaging techniques have significantly enhanced our ability to characterize bone microarchitecture and mechanical properties. Micro-computed tomography (micro-CT) allows high-resolution, three-dimensional quantification of bone structural parameters (Campbell and Sophocleous, 2014; Palacio-Mancheno et al., 2014). This imaging modality, combined with sophisticated image processing, enables detailed analysis of bone architecture (Voide et al., 2006; Burghardt et al., 2011). The micro-CT resolution is sufficient for mapping porosities associated with vascular canals but not for the osteocyte lacuno-canalicular system. Micro-CT resolution can reach 1 μm for *ex vivo* samples using synchrotron radiation (Peyrin et al., 2001; Engelke et al., 1993), while *in vivo* imaging is limited to about 82 μm voxel size due to extensive radiation exposure concerns (Burghardt et al., 2011). Complementing imaging data, mechanical testing on bone samples provides critical information about functional performance (El-Gizawy et al., 2023). These techniques offer volumetric insights unattainable with traditional histology and have become standard tools for pre-clinical assessment of bone architecture during disease progression and treatment (Campbell and Sophocleous, 2014; Bolger et al., 2019). The rabbit femur is particularly suitable for this dual mode characterization; its size permits both detailed imaging and consistent mechanical testing across multiple spatial planes. In contrast, the large diameter and density of human femurs often pose challenges for achieving the resolution and slice uniformity required for high-fidelity 3D structural-mechanical correlation, justifying the use of animal models in this context.

Despite growing interest in bone quality, most existing studies have focused either on global measures of bone mineral density or limited two-dimensional assessments of microstructure, without capturing the full spatial complexity of cortical bone. High-resolution, co-localized datasets that integrate three-dimensional microstructural metrics with corresponding mechanical properties remain scarce, particularly in the femoral shaft - a critical region for load-bearing and orthopedic applications. Furthermore, the radial and longitudinal gradients of features such as porosity and canal diameter is frequently overlooked, despite their likely influence on local mechanical behavior.

Few studies have quantitatively linked these microstructural parameters to compressive modulus in a spatially resolved manner. This study addresses these gaps by performing complete 3D profiling of cortical microarchitecture in the rabbit femoral shaft and systematically correlating these metrics with compressive properties across multiple spatial regions. The findings aim to advance our understanding of structure-function relationship of bone and inform the design of biomimetic materials with spatially optimized mechanical performance.

The primary aim of this study is to conduct a comprehensive three-dimensional characterization of cortical bone microarchitecture and its mechanical properties in the rabbit femoral shaft. Specifically, the objectives are: (1) to quantify gradual changes in porosity, canal diameter, and cortical thickness along both radial and longitudinal axes; (2) to measure compressive modulus in corresponding anatomical regions using mechanical testing on bone slices; and (3) to evaluate the correlations between microstructural parameters and localized mechanical properties. By integrating high-resolution imaging with direct biomechanical assessment, this study seeks to enhance the understanding of bone’s structure-function relationship and provide foundational data for the development of spatially informed designs. It is expected that the comprehensive and detailed characterization of the properties and structure of native bone that are presented here will allow for more realistic functionally graded design and fabrication of synthetic implants and scaffolds for tissue engineering.

## 2 Materials and methods

**FIGURE 1:**
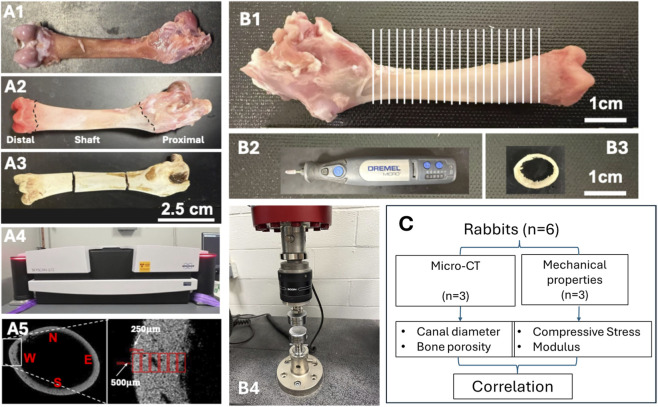
Specimen preparation, instruments, and experimental design. Femur bones **(A1,A2)** were harvested from rabbits, sectioned **(A3)** to fit into the sampling chamber of the micro-CT **(A4)**, and analyzed in terms of pore and canal diameter by collecting data from different regions of the femur **(A5)**. For mechanical characterization, the femurs were sliced in 2 mm thicknesses **(B1)**, processed **(B2)** to obtained uniform slice thickness **(B3)**, and tested under compression **(B4)**. A total of six rabbits were divided equally for micro-CT analysis and mechanical characterization **(C)**.

Specimen preparation, instruments, and experimental design are demonstrated in Figure 1.

### 2.1 Sample preparation

Femoral bones were obtained from commercially sourced rabbits (*Oryctolagus cuniculus*) purchased from a local butcher shop (Jersey City, NJ). These rabbits (male and male) are meat-producing breeds (New Zealand White), slaughtered at an age of approximately 8–12 weeks.

Upon procurement, soft tissues surrounding the femur were carefully removed using a scalpel. The bones were then rinsed with phosphate-buffered saline (PBS) to remove residual debris and immediately stored at −20 °C to preserve structural integrity prior to analysis. All samples were thawed at room temperature for 2 h before any subsequent imaging or mechanical testing. No chemical fixation or decalcification was applied, in order to preserve the native microarchitecture and mechanical properties of the bone tissue.

For imaging (n = 3), following thawing at room temperature, each femur was segmented into three anatomical regions: the distal section containing the femoral condyles, the proximal section containing the trochanters, and the midshaft. The segmentation into distal, midshaft, and proximal regions was performed to analyze region-specific structural variations relevant to different loading conditions and enabled localized structural analysis across the major functional zones of the femur. After dissection, each bone section was thoroughly dried in a vacuum oven at 60 °C for 12 h to ensure complete removal of residual moisture. This dehydration step was essential to prevent any water-related distortion or imaging artifacts and to maintain the structural integrity of the samples during subsequent handling. Care was taken to preserve the native morphology of each segment throughout the drying process.

For the compressive testing, fresh femoral bones were used (n = 3). Mechanical testing was performed on femurs from a separate cohort (additional animals) prepared and tested in the thawed/wet state; thus, mechanical properties were not measured on the dried micro-CT specimens. In this case, only the midshaft portion of the femur was utilized; the distal (condyle) and proximal (trochanter) ends were removed and excluded from analysis. The remaining shaft was evenly sectioned longitudinally into 2.5 mm-thick slices using a low-speed diamond saw (Dremel Multipro Model 395, Racine, WI). The longitudinal position of each slice along the femur was carefully recorded to preserve anatomical context. Each slice was then sanded down to a final thickness of 2.0 ± 0.05 mm using a handheld drill fitted with a sanding attachment (Dremel Micro Model 8050, Mt. Prospect, IL). To avoid thermal damage or structural distortion, sanding was performed under continuous water cooling. This ensured the preservation of native mechanical properties and microarchitecture for downstream analysis.

### 2.2 Micro-CT imaging and analysis

Micro-computed tomography (micro-CT) imaging was conducted on a separate set of dehydrated segments using the SkyScan 1272 system (Bruker microCT, Kontich, Belgium) equipped with a Photonic Science PS52 detector. Imaging was performed at a tube voltage of 100 kV and a current of 94 µA. A dual-filter configuration consisting of a 0.5 mm aluminum filter and a 0.038 mm copper filter was used to reduce beam hardening effects. The exposure time was set to 4,000 ms per projection, with frame averaging set to three frames per projection. Scanning was carried out over a full 360° rotation with a rotation step of 0.4°, yielding a total of 900 projections per scan. The effective voxel size of the reconstructed images was 5.8 µm. Image reconstruction was performed using NRecon software (version 2.2.0.6, Bruker) with GPU acceleration enabled. Binary segmentation for porosity quantification was performed in CTAn (CT Analyser v1.23.0.1) using global thresholding with a lower gray threshold of 80 and upper gray threshold of 255 (applied consistently across the analyzed images/ROI). After thresholding, a despeckle filter was applied to remove isolated segmentation artifacts by eliminating white speckles in 2D smaller than 25 pixels. Porosity was computed as the void volume fraction within each ROI. Ring artifact correction was applied at level 12. Beam hardening correction and image smoothing were not applied in order to preserve the original grayscale distribution for subsequent quantitative analysis.

For the microstructural analysis, the 3D reconstructed model was sliced at 1 mm intervals. On each slice, the region of interest (ROI) was defined as a rectangular window measuring 500 × 250 μm^2^, positioned from the outer cortical surface toward the inner cortex. Measurements were taken at four orthogonal orientations—anterior, posterior, medial, and lateral—within each transverse micro-CT slice. These directional measurements were averaged to represent the radial structural profile at each longitudinal position, with data points collected at 250, 500, 750, 1,000 μm, etc., from the outer surface, continuing inward until no bone material remained within the ROI. Quantitative structural parameters extracted from each ROI included porosity (%), canal diameter (µm), and bone thickness (mm). All measurements were performed on the entire bone area within the defined ROI, restricted to the shaft region and excluding the distal and proximal femur, using calibrated image analysis protocols.

For the distal femur, proximal femur, and shaft segments, canal diameters were binned into defined size ranges, and the corresponding volume occupied by canals in each range was computed. The canal diameter distribution was expressed as the percentage volume of canals within a given diameter range relative to the total canal volume in the analyzed bone segment. This analysis allowed for comparative evaluation of canal architecture across distinct anatomical regions of the femur. Structural parameters derived from micro-CT imaging guided the interpretation of mechanical responses measured during subsequent compression tests.

### 2.3 Compression testing

Compression testing was performed on bone slices using a uniaxial testing system (Instron 5980, Norwood, MA). Each specimen had a thickness of 2.0 ± 0.05 mm and was derived from the femoral shaft region, excluding the distal and proximal ends. Prior to testing, all specimens were visually inspected to ensure parallel surfaces and absence of visible defects. Tests were conducted at room temperature with a crosshead displacement rate of 1 mm/min. No preload was applied. Load and displacement data were recorded continuously during each test and used to generate stress–strain curves.

Three mechanical parameters were extracted from the data: modulus 1, defined as the slope of the stress–strain curve in the first linear region; modulus 2, defined as the slope of the stress–strain curve in the second linear region; and maximum compressive strength, defined as the peak stress before failure. A total of three rabbits were used for this analysis, with multiple slices tested per animal to capture gradients along the femoral shaft. The exact number of slices per sample was recorded based on their anatomical position.

### 2.4 Statistical analysis

Statistical analyses were performed to evaluate the relationship between porosity and Modulus. The Pearson correlation coefficient (r) was used to measure the strength and direction of the linear association between the variables. Data were analyzed both as individual experimental replicates (n = 3) and as a pooled dataset for means to identify consistent trends. All statistical tests were two-tailed, with the alpha level for significance set at p < 0.05. Analysis was conducted using Excel.

## 3 Results

### 3.1 Cortical thickness variation along the femur

**FIGURE 2:**
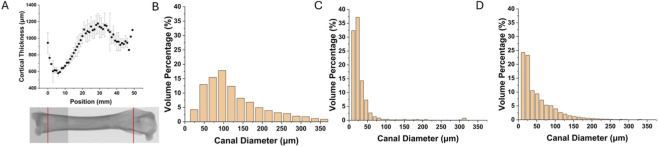
Change in cortical thickness with respect to distance from distal femur **(A)**, and histogram of volume percent of the canal diameter for the distal femur **(B)**, shaft **(C)**, and proximal femur **(D)**.

To understand load-bearing adaptations, we first assessed variations in cortical bone thickness along the femoral shaft. Bone thickness was measured at 1 mm intervals along the longitudinal axis of the femur, starting from the distal end. The results demonstrated a non-uniform distribution of bone thickness along the length of the bone. As shown in Figure 2A, cortical thickness first decreased and then increased from the distal femur toward the midshaft, reaching a maximum in the central portion of the shaft, followed by a gradual decrease toward the proximal end.

### 3.2 Regional canal diameter distribution

Canal diameter distributions were quantified for the distal femur, shaft, and proximal femur to evaluate how intracortical vascular space varied regionally. Figures 2B–D show the volume percentage of canals distributed across diameter bins ranging from 0 to 375 µm. In the distal femur (Figure 2B), canal volume was primarily concentrated in the 50–200 µm range. The shaft region (Figure 2C) exhibited a narrower distribution, with the majority of canal volume confined to the 0–50 µm range. In the proximal femur (Figure 2D), canal diameters were mostly concentrated within the 0–100 µm range, with minimal volume beyond this threshold and a maximum diameter of approximately 250 µm.

The observed variability in canal diameter distributions across anatomical regions supports the selection of a fixed region of interest measuring 500 × 250 μm^2^ for micro-CT analysis. As demonstrated in Figure 2, canal diameters in the femoral shaft are predominantly below 50 μm, while the proximal and distal femur contain a broader distribution with canal diameters extending beyond 100 μm, and in some cases exceeding 250 μm, particularly in the distal region.

The chosen ROI dimensions allow for the consistent capture of the majority of canal structures present across all regions, while maintaining sufficient resolution for small-diameter canals in the shaft. Although some of the largest canals may partially fall outside the defined ROI, especially in the distal femur, the selected size offers a practical balance between resolution and anatomical coverage for comparative analysis. The distinct regional shifts in canal diameter suggested that void morphology varies not only in magnitude but also in spatial distribution; therefore, the following subsection maps porosity and mean canal diameter along both the femur’s length and its cortical thickness to integrate size metrics with overall void fraction.

### 3.3 Radial gradients in porosity and canal diameter

**FIGURE 3:**
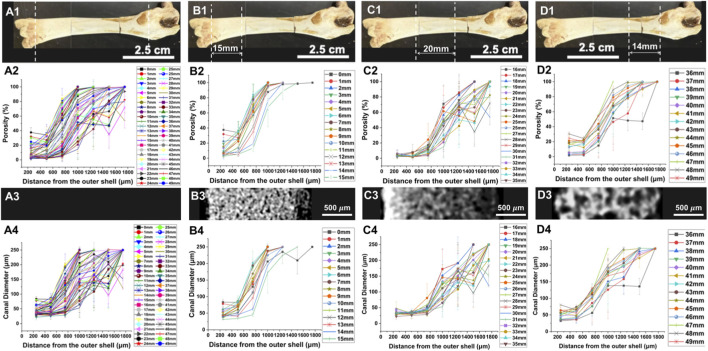
Change in porosity and canal diameter with respect to distance from the outer surface for the entire femur **(A)**, distal end **(B)**, shaft **(C)**, and proximal end **(D)**. The femur was scanned at 1 mm intervals in the longitudinal direction for the segmented bones **(B1,C1,D1)** and merged to report the variation over the entire bone **(A1)**. Porosity and canal diameter variation were measured from the scanned slices for the segmented bones (**(B2,C2,D2,B4,C4,D4)**, respectively), and overlaid to obtain porosity change and canal diameter change over the entire scanned region (**(A2,A4)**, respectively). Representative micro-CT images were provided from each segmented region **(B3,C3,D3)**.

Porosity and canal diameter were analyzed along the radial direction (distance from the outer surface) of the femur using reconstructed micro-CT slices at 1 mm intervals in the longitudinal direction. Figure 3A1 presents a representative image of the full femur, with white dotted lines indicating the portion of the bone of which the porosity and canal diameter were measured as a function of the distance from the outer surface. Similar regional indicators are shown for the distal, shaft, and proximal segments in Figures 3B1, 3C1, 3D1, respectively.

Regional measurements are detailed in Figures 3B2–D2 for porosity and Figures 3B4–D4 for canal diameter. In the distal femur, shaft, and proximal region, the porosity gradually increased from the outer surface towards the core of the bone. Representative transverse micro-CT images from each region are shown in Figures 3B3, 3C3, 3D3. The distal (Figure 3B3) and proximal (Figure 3D3) regions show more porous bone with visibly larger and more numerous canals, while the shaft region (Figure 3C3) displays a denser structure with fewer and smaller canals, consistent with the quantitative porosity and canal diameter data.

### 3.4 Longitudinal microstructural gradients

**FIGURE 4:**
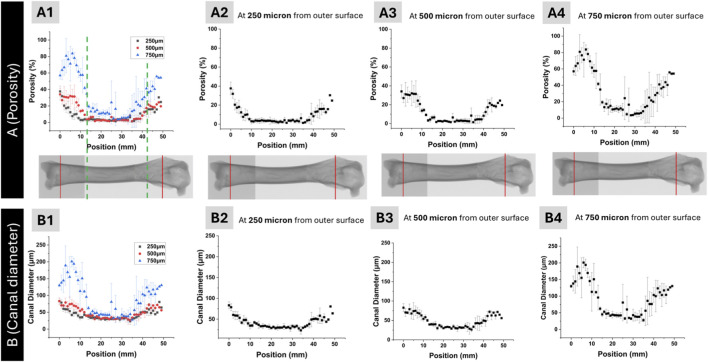
Porosity **(A)** and canal diameter **(B)** distribution in the femur as a function of longitudinal distance. Overlaid porosity distribution at 250, 500, and 750 μm distance from the outer surface **(A1)**, and porosity distributions at 250 μm **(A2)**, 500 μm **(A3)**, and 750 μm **(A4)**. Overlaid canal diameter distribution at 250, 500, and 750 μm distance from the outer surface **(B1)**, and canal diameter distributions at 250 μm **(B2)**, 500 μm **(B3)**, and 750 μm **(B4)**. The distal and proximal regions, where porosity is ∼40% and above are indicated by dotted green lines in **(A1)**.

The data presented in Figure 4 were extracted from the same dataset used in Figure 3. For this analysis, porosity and canal diameter values were obtained specifically at 250, 500, and 750 µm from the outer surface and plotted against the longitudinal position along the femur.

The longitudinal overall trend in porosity is shown in Figure 4A for the entire bone length. Porosity values were higher in the distal and proximal regions, with values between 40%–80%, and lower in the shaft, with values between 5%–35%. This behavior is more pronounced in the outer regions of the bone as measured at 250 μm and 500 μm distance from the outer surface. Although the transition from highly porous trabecular (distal and proximal regions) to low porosity cortical (shaft region) demonstrates gradual change, a line of separation approximating the location of transition from cortical to trabecular bone was placed in Figure 4A1. A similar trend was observed for canal diameter (Figure 4B), with reduced values in the shaft and elevated diameters toward both ends of the femur.

Figure 4A1 presents the overlay of porosity distributions at these three distances. The profiles shift with increasing distance, showing lower porosity at 250 µm and elevated porosity at greater distances from the outer surface. Individual porosity distributions at 250, 500, and 750 µm are shown in Figures 4A2–A4, respectively. Porosity at 250 µm was relatively low and exhibited a narrow distribution, while porosity at 500 and 750 µm increased in both magnitude and spread, indicating a gradient in structural void content across the cortical thickness.

Canal diameter distributions across the same distances from the outer surface are shown in Figure 4B1 as an overlay, with individual profiles at 250, 500, and 750 µm presented in Figures 4B2–B4, respectively. At 250 μm, canal diameter along the femur was concentrated within the 0–75 µm range, with limited variation between anatomical regions. At 500 μm, the overall range remained similar, with most values still falling below 75 μm; however, slight local increases were observed in the distal and proximal regions. In contrast, at 750 μm, canal diameter increased noticeably in the distal and proximal regions, reaching values up to 200 μm, while the shaft remained confined to a lower range comparable to that at 250 μm and 500 µm. These data indicate that substantial enlargement of canals with respect to longitudinal position primarily occurs at greater distances from the outer surface.

**FIGURE 5:**
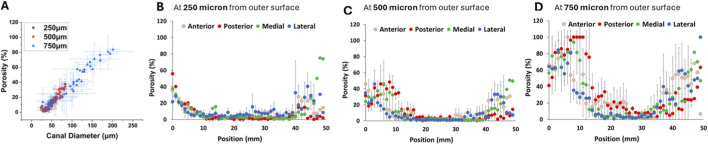
**(A)** Correlation of porosity and canal diameter at 250, 500, and 750 µm distance from the outer surface. Gradients of canal diameter at Anterior, Posterior, Medial and Lateral directions over the length of the femur at **(B)** 250 µm, **(C)** 500 µm, and **(D)** 750 µm from the outer surface.

Figure 5A presents the correlation between porosity and canal diameter at 250, 500, and 750 µm from the outer surface. This plot was generated using the same dataset shown in Figure 4 by pairing porosity and canal diameter values at corresponding positions along the femur. A linear relationship was observed at each distance, with comparable slopes across the three curves. While the overall trend was consistent, the curve at 750 µm extended to higher values than those at 250 and 500 µm. Specifically, porosity reached approximately 80% and was associated with canal diameters up to 200 µm in the distal and proximal regions. This extended range at 750 µm reflects the localized increase in both canal size and void volume deeper within the bone at those positions.

Quantifying how porosity and canal diameter evolve from the outer to the inner cortex raised the question of whether these microstructural changes translate into local mechanical behavior; accordingly, the next section relates the radial data to position-matched compressive moduli.

The position-dependent (Anterior, Posterior, Medial and Lateral) gradients of porosity were plotted in Figures 5B–D at 250, 500 and 750 µm from the outer surface, respectively. Generally, the posterior and medial quadrants consistently exhibit higher porosity compared to the anterior and lateral regions across all depths (Figure 5D). Specifically, at the 750 µm depth, the posterior region shows very high porosity values at several longitudinal positions, whereas the lateral and anterior regions maintain relatively lower, though still elevated, porosity levels.

### 3.5 Mechanical properties and microstructure correlation

**FIGURE 6:**
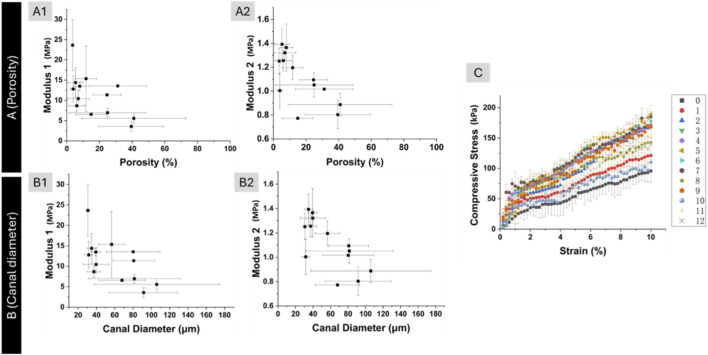
Correlation of modulus 1 and porosity **(A1)**, modulus 2 and porosity **(A2)**. Correlation of modulus 1 and canal diameter **(B1)**, modulus 2 and canal diameter **(B2)**. Compressive stress and strain curve for mechanical compression test **(C)**. In **(C)**, the legend shows the number of sections with ∼2 mm thickness.

Relationships between microstructural parameters and mechanical properties were evaluated to confirm the role of microarchitecture in stiffness. Correlations between bone microstructure and compressive properties were assessed by relating porosity and canal diameter to modulus 1 and modulus 2. Figure 6A1 shows the relationship between porosity and modulus 1. As porosity increased from approximately 5%–40%, modulus 1 decreased from about 15 MPa to 5 MPa. A similar inverse correlation was observed between porosity and modulus 2 in Figure 6A2. Over the same porosity range (5%–40%), modulus 2 declined from approximately 1.4 MPa–0.7 MPa.

A Pearson correlation analysis was conducted to assess the association between porosity and Modulus 1. Across the combined dataset, we observed a very weak negative correlation for the pooled data (r = −0.158), p = 0.405). Analysis of a specific subset (n = 3) of 13 data pairs similarly yielded a non-significant, weak negative relationship (r = −0.116, p = 0.706). The same analysis was performed on porosity and modulus 2. We observed a very weak negative correlation for the pooled data (r = −0.214, p = 0.305). Analysis of a specific subset of 13 data pairs similarly yielded a non-significant, weak negative relationship (r = −0.116, p = 0.706). Apparently, the relationship between modulus and porosity is non-linear.

Figures 6B1,B2 show the correlations between average canal diameter and modulus 1 and modulus 2, respectively. As canal diameter increased from approximately 20 µm–110 μm, both modulus 1 and modulus 2 exhibited decreasing trends. Modulus 1 declined from around 15 MPa to approximately 5 MPa over this range. Similarly, modulus 2 decreased from about 1.4 MPa to 0.7 MPa. Although the data were more scattered compared to the porosity-based correlations, the overall negative association between canal diameter and compressive stiffness was consistently observed in both metrics.

Figure 6C presents compressive stress–strain curves for all femur slices subjected to mechanical testing. The legends indicate the slice numbers, which correspond to their longitudinal positions along the femur. Each curve exhibited two distinct linear regions. The slope of the first linear region was used to calculate modulus 1, while the slope of the second linear region was used to calculate modulus 2, as described in the methods section. The presence of two linear regions across all tested slices demonstrates the consistency of this mechanical behavior throughout the femur. While the point correlations confirm that stiffness diminishes with increasing void volume and canal size, a continuous profile is required to visualise how structural and mechanical properties co-vary along the entire femoral shaft; the subsequent analysis provides this integrated longitudinal map.

### 3.6 Longitudinal variation of mechanical and structural properties

**FIGURE 7:**
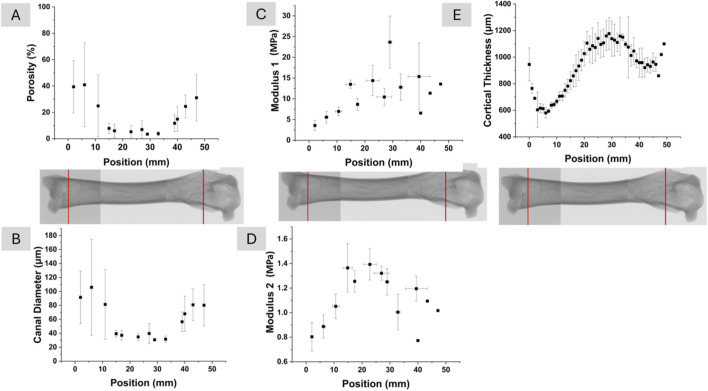
Porosity **(A)** and canal diameter **(B)** distribution in the femur as a function of the position. Correlation of modulus 1 and position **(C)**, modulus 2 and position **(D)**. Longitudinal variation in bone thickness **(E)**.

Finally, structural and mechanical profiles were integrated to reveal spatial coordination along the femur shaft. Figure 7 presents the variation of microstructural and mechanical parameters along the longitudinal axis of the femur. Average porosity and average canal diameter trends are shown in Figures 7A,B, respectively. Average porosity values were lowest in the shaft region, ranging between 2% and 5%, and increased progressively toward the distal and proximal ends, reaching up to approximately 40%. Average canal diameter exhibited a similar longitudinal pattern, with values between 30 and 40 µm in the shaft and increasing to as high as 110 μm at the bone ends. These trends are consistent with the regional and radial analyses presented in earlier sections.

Figures 7C,D show the variation of modulus 1 and modulus 2 along the longitudinal axis of the femur. It is noted here that modulus 1 and modulus 2 are the apparent moduli influenced by the highly porous structure of the bone. Both modulus values peaked in the shaft region and decreased toward the bone ends. Modulus 1 ranged from approximately 5 MPa at the distal end (0 mm), increased to around 25 MPa in the central shaft, and then declined to about 10 MPa at the proximal end (50 mm). Modulus 2 followed a similar trend, rising from approximately 0.7 MPa at the distal end to 1.4 MPa in the shaft and decreasing to around 1.0 MPa at the proximal end. These longitudinal profiles reflect a central zone of greater compressive stiffness, consistent with the lower porosity and smaller canal diameter observed in the shaft region.

Figure 7E shows the longitudinal variation in bone thickness. The overall pattern of bone thickness closely follows the trends observed for modulus 1 and modulus 2 in Figures 7C,D. Bone thickness increased from the distal end toward the shaft and then gradually decreased toward the proximal end. This progression corresponds with the region of highest compressive stiffness and lowest porosity along the shaft, as shown in Figures 7A,B. The spatial alignment of bone thickness with structural and mechanical parameters suggests a consistent anatomical transition along the femur.

## 4 Discussion

Our micro-CT analysis revealed that cortical thickness was highest at the femoral mid-shaft and tapered toward both proximal and distal ends. This region is known to carry the largest bending and axial loads during routine gait, so the extra thickness likely reinforces the bone against fracture (Someya et al., 2020; Johannesdottir et al., 2011; Nawathe et al., 2015). The thinner cortices near the bone ends may represent an anatomical compromise that provides space for blood vessels and allows a gradual transition to trabecular bone at the metaphysis (Gabet et al., 2008; Ellis et al., 2011). Previously, Pazzaglia et al. studied New Zeeland rabbits and found no significant difference between the cortical thicknesses of distal and proximal femur (Pazzaglia et al., 2014). Overall, these results support that region-specific variations in cortical thickness are a key structural factor governing the mechanical competence of long bones.

Following the thickness analysis, micro-CT imaging showed that vascular canals were widest in the distal cortex (50–200 µm), smallest at the mid-shaft (<50 µm), and intermediate proximally. This regional pattern means that screws or rods of implants pressed into the distal cortex engage a more porous, less stiff substrate than at the shaft, creating a stiffness mismatch that has been linked to early screw loosening and plate-end failure in clinical studies (BOBYN et al., 1992; Bobyn et al., 1981; Wang et al., 2020). A mismatch between the implants and the native bone tissue could additionally place unbalanced loads over the knee joint, further damaging the joint components, including the anterior cruciate ligament (ACL). This unbalanced loading may tear/rupture the ACL, leading to joint instability (Kadyr et al., 2024; Smatov et al., 2023; Ad et al., 2022). Modern plates and scaffolds are usually uniform in density; reproducing the larger distal canals while preserving the tighter mid-shaft architecture could reduce such failures and speed blood-vessel ingrowth during regeneration. Our quantitative canal-diameter map therefore offers practical dimensions for graded implants and additively manufactured scaffolds that aim to match native cortical structure along the bone length.

Both longitudinally and radially, porosity and canal diameter increased notably toward the femoral ends. Porosity and mean canal diameter were roughly three-times higher at the femoral ends (∼28% porosity; ∼110 µm canals) than at the mid-shaft (∼9%; ∼40 µm). These longitudinal gradients lower stiffness where loads are smaller and boost blood supply and remodeling capacity near joints, but it also means that uniform metal plates or nails over-stiffen the porous ends and can concentrate stresses that lead to screw loosening or end-fracture. Additively manufactured implants and scaffolds that grade pore size—larger at the ends, denser at mid-shaft—would better share load with the host bone (Velioglu et al., 2019) and speed vascular invasion during healing (Li et al., 2020; Gallab et al., 2024; Jin et al., 2025).

Moving from lengthwise patterns to the radial direction, porosity and canal diameter rose steadily from the periosteal surface toward the medullary cavity, reaching values of about 80% porosity and ∼200 µm canals in the inner cortex of distal and proximal regions. The radial gradient in porosity and canal diameter, increasing notably from the periosteal surface inward, indicates structural adaptation that might prevent full-thickness fracture propagation (Uhthoff et al., 2006). Layered implants or scaffold struts that are denser externally and more open internally would better match native load transfer and speed marrow-side vascular invasion (Di Luca et al., 2016a); present devices rarely achieve such through-thickness grading.

Long bones exhibit functional adaptation to bending, meaning the medial (compressive) and lateral (tensile) cortices often have different microstructural signatures. In this study, the primary figures report an averaged ‘global’ profile to emphasize the robust longitudinal and through-thickness trends; however, direction-resolved analysis is included to show whether asymmetry exists and to prevent clinically relevant gradients from being obscured by averaging. The experimental results should also be interpreted in the context of the femur’s functional adaptation to asymmetrical loading, where the medial and lateral cortices develop distinct microstructural signatures. During gait, the medial cortex is subjected to high compressive loads, while the lateral cortex predominantly resists tensile stress. Because bone is naturally weaker in tension than in compression, the lateral cortex is more sensitive to structural failure. Our findings show that even at 250 µm, the lateral cortex maintains lower porosity compared to the medial region in certain longitudinal positions, a microstructural strategy likely intended to maximize tensile strength. Furthermore, research indicates that tensile-stressed cortices often exhibit greater porosity and different osteonal patterns—such as fewer, larger osteons—compared to compressive cortices, which typically favor denser, interstitial bone to maintain stiffness. The high porosity observed at 750 µm in the lateral region (Figure 5D) could therefore represent a critical structural vulnerability, as the combination of high tensile stress and increased porosity exponentially reduces the bone’s fracture toughness and bending resistance.

Building on the spatial gradients already described, our findings showed that both modulus 1 (initial modulus) and modulus 2 (secondary modulus) dropped wherever porosity or mean canal diameter increased: denser regions with smaller canals were stiffer, while more porous regions with larger canals were more compliant. To our knowledge, this is the first study to chart modulus 1 and modulus 2 against porosity and canal diameter across the full shaft of the rabbit femur model, giving a practical way to estimate position-specific stiffness with regard to microstructural properties. Together, these findings support microarchitecture, rather than bulk mineral density, governs cortical mechanics and supply reference values for future comparative or modelling work (Augat and Schorlemmer, 2006; Seeman, 2017; Renders et al., 2007; Schneider et al., 2013; Uniyal et al., 2021).

Thermal changes in collagen structure have been documented in the literature, with denaturation/shrinkage behavior depending on tissue state and conditions (Zhang et al., 2020; Surowiec et al., 2022). In our study, the structural metrics extracted by micro-CT (porosity and canal diameter) quantify the mineralized cortical void network geometry, which is less sensitive to hydration than mechanical properties. Importantly, prior work has reported no measurable difference in intracortical porosity by micro-CT between wet and partially dried cortical bone specimens under low-energy drying conditions (Nyman et al., 2013). Nonetheless, drying at 60 °C could, in principle, introduce microcracks or alter apparent pore morphology. Therefore, micro-CT and mechanical testing were performed on different sample sets, and it is clarified here that “dry-state” micro-CT was used for geometric microarchitecture mapping, whereas mechanical testing was performed in the thawed (hydrated) state to better represent functional behavior.

Our integrated analysis clearly showed mid-shaft as the zone of highest structural robustness and stiffness. Modulus 1 and modulus 2 were highest at the femoral mid-shaft and declined by roughly 40% toward both distal and proximal ends, matching the longitudinal shifts in thickness, porosity, and canal size described earlier. This alignment shows that the femur gradually softens where loads are lower, a strategy that avoids stress peaks and saves material. Conventional plates and intramedullary rods are uniform in stiffness; by over-supporting the naturally stiff mid-shaft and under-matching the more flexible ends, they can shift load to screw holes and promote plate-end failure (Wang et al., 2020; Ebrahi et al., 2012). Functionally graded implants that are rigid at the mid-shaft and progressively more compliant toward the ends would better track the native modulus profile as would additively manufactured scaffolds printed with a similar stiffness gradient to guide regeneration (Oshkour et al., 2013; Ataollahi Oshkour et al., 2015).

The spatial maps of bone thickness, porosity, canal diameter, and modulus presented here offer quantitative targets for the next-generation of total hip and knee replacement devices and for tissue-engineered osteochondral grafts (Erisken et al., 2008a; Yildirim et al., 2023; Effanga et al., 2024; Zhao et al., 2025). Metallic stems, cups, and tibial trays manufactured from uniform Ti-6Al-4V or Co–Cr alloys typically possess elastic moduli far exceeding those of host bone, particularly at the metaphyseal ends, thereby redistributing load and promoting stress shielding (Wang et al., 2020). By documenting a pronounced axial drop in cortical stiffness from the femoral mid-shaft toward both the distal and proximal regions concomitant with a marked rise in porosity and canal diameter, the present study defines the magnitude and length-scale of the gradient that graded stems or trays must reproduce to maintain physiological load transfer and mitigate aseptic loosening.

Axial grading alone, however, cannot resolve interfacial mismatch if radial heterogeneity is ignored. The observed progressive increase in porosity and canal diameter from the outer cortex toward the medullary cavity demonstrates that cortical bone functions as a dense shell surrounding a more compliant, highly vascularized core. Functionally graded scaffolds/grafts (Effanga et al., 2024; Zhao et al., 2026; Kadyr et al., 2025) with seamless gradient that emulate this radial transition can therefore couple high interfacial stiffness and wear resistance at the surface with enhanced vascular and cellular infiltration internally (Li et al., 2024; Di Luca et al., 2016b). Such bimodal designs align with emerging porous-metal and polymer-ceramic composites that report accelerated osseointegration when inner canal diameters exceed one hundred micrometers (Karageorgiou and Kaplan, 2005).

Beyond empirical design, the strong relationships quantified here between local modulus and structural metrics such as porosity and canal diameter establish data-driven rules for assigning position-dependent material properties in the analysis of implants and grafts. Incorporating this relationship allows simulations to capture the gradual load hand-off from implant to host tissue, providing a foundation for optimizing regional implant density, structure thickness, and material composition prior to manufacturing. Earlier, a strong dependence of modulus on porosity was determined at smaller length scales (Kaya et al., 2017); however, in our study we could not find a strong and significant correlation.

Our approach combined high micro-CT with location-matched compression tests, allowing us to link local modulus values to canal diameter and porosity with millimeter-scale precision. Although the rabbit model provides valuable insights due to practical advantages, higher turnover rates and anatomical differences limit direct translation of absolute thresholds to human bones, highlighting the need for cautious extrapolation. Additional limitations include the modest sample size, removal of periosteum during preparation (which may alter hydration), and the use of uniaxial compression rather than bending, all of which could influence absolute modulus values. Even so, the thorough 3D study and profiling of rabbit femur microstructural and biomechanical properties strengthen confidence in the positional patterns reported here and provide a clear baseline for future graded-implant and scaffold studies.

Future studies should extend these findings to larger animal or human cadaveric models, evaluate changes with aging and disease, and use these refined data sets to directly guide the design of implants and scaffolds optimized for clinical translation. Second, longitudinal micro-CT and matched mechanical testing in ageing or osteoporotic rabbits could track how these spatial patterns evolve with disease or time and establish thresholds for intervention. Third, the quantitative maps of thickness, porosity, canal size, and modulus reported here can be integrated into the design loop for implants and scaffolds for tissue engineering, enabling graded biomaterials that better match native stiffness and vascular requirements than today’s uniform devices. Functionally graded materials (FGMs), i.e., composites characterized by spatially varying properties that transition continuously or discretely across dimensions (Schneider et al., 2013; Uniyal et al., 2021; Pompe et al., 2003; Mikos et al., 2006; Leong et al., 2008; Puppi et al., 2010) can be used. Such materials can be designed to address the limitations inherent in homogeneous systems, particularly at interfaces where abrupt property differences can lead to mechanical failure or poor biological integration (Uniyal et al., 2021; Wegrzyn et al., 2010). FGMs enable gradual changes in composition, microstructure, or porosity, facilitating improved load transfer, stress distribution, and material compatibility in structural and biomedical applications (Schneider et al., 2013; Lloyd et al., 2015; Feng and Todoh, 2021; Puppi et al., 2010).

The osteochondral interface, which connects articular cartilage to the underlying subchondral bone, is a prototypical example of a biological FGM. It transitions from a viscoelastic, avascular cartilage layer to a highly mineralized bone layer, reflecting a continuous increase in mechanical stiffness across the interface (Wang et al., 2020; Abbasi et al., 2020). Mimicking this gradient would be essential to engineering grafts that perform comparably to native tissue under physiological loading conditions (Karageorgiou and Kaplan, 2005; Krishna et al., 2007).

An additional recent focus in biomaterials research has been on integrating fabrication techniques that are industrially relevant, such as extrusion-based bioprinting, hybrid extrusion and electrospinning, coextrusion for multiple layers, hybrid extrusion and spiral winding, melt electrowriting to develop FGMs with fine-tuned gradients in mineral content and mechanical properties (Erisken et al., 2008a; Erisken et al., 2008b; Chung et al., 2018; Tourlomousis et al., 2017; Tourlomousis et al., 2019; Senturk-Ozer et al., 2016; Ozkan et al., 2009; Ozkan et al., 2010; Erisken et al., 2010; Ergun et al., 2011a; Ergun et al., 2011b; Ergun et al., 2012a; Ergun et al., 2012b; Chen et al., 2013). Bioartificial scaffolds could also serve as useful biomaterials due to the preserved structure of the tissue (Seitzhaparova et al., 2025). Such technologies have the potential to facilitate seamless transitions between distinct biomaterial phases and can be instrumental in the development of osteochondral scaffolds that closely mimic the native interface (Lu et al., 2010; Erisken et al., 2011; Seidi et al., 2011).

Lastly, while the rabbit model provides rapid healing and tractable size, its higher turnover rate and thinner cortex mean that absolute thresholds may differ from those in larger animals or in humans. Extending the present mapping to ovine or cadaveric femoral shafts, and tracking how the gradients shift with age, disease, and cyclic loading, will refine the design window for clinical devices. Integrating our micro-CT–derived modulus relationships into finite-element simulations and iterative implant prototyping will further accelerate translation. Additionally, micro-CT imaging was conducted on dehydrated specimens (60 °C, 12 h), whereas mechanical testing was performed a separate cohort. Because hydration strongly affects bone mechanical behavior, modulus values are not directly comparable across hydration states. While the micro-CT analysis targets the mineralized void network geometry (porosity/canal diameter), future studies will include paired scans of wet/chemically fixed *versus* dried specimens to directly verify that the dehydration protocol does not introduce microcracking or bias porosity quantification. Furthermore, the lacunocanalicular system (LCS) is the primary site for osteocyte mechanosensing and plays a critical role in bone biomechanics *via* fluid shear stress perception. Here we focused mainly on the vascular canal system due to following considerations: Firstly, the primary goal of this study was to define structural benchmarks for functionally graded scaffolds and macroscopic implants. While the LCS is essential for cellular signaling, the macroscopic stiffness and bulk permeability of cortical bone, which govern load distribution and initial vascular ingrowth in regenerative medicine, are primarily dictated by the larger-scale cortical porosity and vascular canal architecture (∼40–200 µm in our study). Replicating these larger gradients is the first essential step in creating “native-like” stiffness transitions in synthetic implants. Also, our study utilized high-resolution micro-computed tomography (micro-CT) to quantify microgradients. While this technology is excellent for capturing vascular canals (typically 50–100 µm), the canaliculi themselves are sub-micron structures requiring advanced techniques like Synchrotron Radiation CT or Confocal Laser Scanning Microscopy, which were beyond the scope of this baseline structural study. Finally, it is important to note that the Haversian canals act as local “sinks” for the fluid flow that moves through the LCS. Therefore, by mapping the longitudinal and radial gradients of these larger canals, we have implicitly provided a framework for the boundary conditions that govern LCS fluid dynamics and subsequent mechanotransduction. In this regard, while our study focuses on the vascular scale necessary for scaffold design, the interplay between these macro-pores and the microscopic lacunocanalicular mechanosensing network is a vital area for future multi-scale modeling.

## 5 Conclusion

This work set out to create a detailed, spatially resolved picture of structure–function relationships along the rabbit femoral shaft and to translate those insights into design criteria for next-generation orthopedic implants and tissue-engineered scaffolds. By pairing high-resolution micro-CT imaging with location-matched compression testing, we mapped bone thickness, porosity, vascular-canal diameter, and local modulus at millimeter-scale precision, producing, to the best of our knowledge, the first full-wall dataset of its kind for any long bone in the rabbit model.

Three consistent patterns emerged. First, bone thickness peaked at mid-shaft and tapered toward both ends, aligning with the region of greatest habitual bending and axial load. Second, porosity and mean canal diameter rose not only from shaft to ends but also from the outer surface toward the medullary cavity, creating combined longitudinal and radial gradients in void fraction. Third, both the initial and post-yield moduli fell wherever porosity or canal size increased, demonstrating that microarchitecture—rather than bulk mineral density—governs cortical stiffness. Together, these findings reveal a shaft that is simultaneously thick, dense, and stiff, flanked by ends that are thinner, more porous, and mechanically compliant. Translating these observations to practice, the numeric profiles reported here offer concrete targets for functionally graded plates, intramedullary rods, and additively manufactured scaffolds. Implants that preserve a stiff mid-shaft segment while gradually reducing stiffness and increasing permeability toward the bone ends—and toward the marrow side—could mitigate screw loosening, stress shielding, and delayed union. Likewise, scaffolds patterned with smaller, load-bearing pores at mid-shaft and larger, angiogenic pores distally and medially should better balance mechanical support with vascular access during regeneration.

## Data Availability

The original contributions presented in the study are included in the article/supplementary material, further inquiries can be directed to the corresponding authors.

## References

[B1] Abbasi N. Hamlet S. Love R. M. Nguyen N. T. (2020). Porous scaffolds for bone regeneration. J. Sci. Adv. Mater. Devices 5, 1–9. 10.1016/j.jsamd.2020.01.007

[B2] Adeoye A. O. Mukasheva F. Smatov S. Khumyrzakh B. Kadyr S. Shulgau Z. (2022). A biomimetic synthetic nanofiber-based model for anterior cruciate ligament regeneration. Front. Bioeng. Biotechnol. 10, 969282. 10.3389/fbioe.2022.969282 36394020 PMC9644221

[B3] Ataollahi Oshkour A. Pramanik S. Mehrali M. Yau Y. H. Tarlochan F. Abu Osman N. A. (2015). Mechanical and physical behavior of newly developed functionally graded materials and composites of stainless steel 316L with calcium silicate and hydroxyapatite. J. Mech. Behav. Biomed. Mater. 49, 321–331. 10.1016/j.jmbbm.2015.05.020 26072197

[B4] Augat P. Schorlemmer S. (2006). The role of cortical bone and its microstructure in bone strength. Age Ageing 35, ii27–ii31. 10.1093/ageing/afl081 16926200

[B5] Bagi C. M. Berryman E. Moalli M. R. (2011). Comparative bone anatomy of commonly used laboratory animals: implications for drug discovery. Comp. Med. 61, 76–85. 21819685 PMC3060425

[B6] Bobyn J. D. Pilliar R. M. Cameron H. U. Weatherly G. C. (1981). Osteogenic phenomena across endosteal bone-implant spaces with porous surfaced intramedullary implants. Acta Orthop. Scand. 52, 145–153. 10.3109/17453678108991775 7246092

[B7] Bobyn J. D. Mortimer E. S. Glassman A. H. Engh C. A. Miller J. E. Brooks C. E. (1992). Producing and avoiding stress shielding. Clin. Orthop. Relat. Res. 274, 79–96. 10.1097/00003086-199201000-00010 1729025

[B8] Bolger M. W. Romanowicz G. E. Kohn D. H. (2019). Advancements in composition and structural characterization of bone to inform mechanical outcomes and modeling. Curr. Opin. Biomed. Eng. 11, 76–84. 10.1016/j.cobme.2019.09.011 32864522 PMC7450708

[B9] Boskey A. L. Imbert L. (2017). Bone quality changes associated with aging and disease: a review. Ann. N. Y. Acad. Sci. 1410, 93–106. 10.1111/nyas.13572 29265417 PMC5774017

[B10] Brandi M. L. (2009). Microarchitecture, the key to bone quality. Rheumatol. (United Kingdom) 48, iv3–iv8. 10.1093/rheumatology/kep273 19783591

[B11] Burghardt A. J. Link T. M. Majumdar S. (2011). High-resolution computed tomography for clinical imaging of bone microarchitecture. Clin. Orthop. Relat. Res. 469, 2179–2193. 10.1007/s11999-010-1766-x 21344275 PMC3126972

[B12] Cai X. Brenner R. Peralta L. Olivier C. Gouttenoire P. J. Chappard C. (2019). Homogenization of cortical bone reveals that the organization and shape of pores marginally affect elasticity. J. R. Soc. Interface 16, 20180911. 10.1098/rsif.2018.0911 30958180 PMC6408344

[B13] Campbell G. M. Sophocleous A. (2014). Quantitative analysis of bone and soft tissue by micro-computed tomography: applications to *ex vivo* and *in vivo* studies. Bonekey Rep. 3, 564. 10.1038/bonekey.2014.59 25184037 PMC4140449

[B14] Chen X. Ergun A. Gevgilili H. Ozkan S. Kalyon D. M. Wang H. (2013). Shell-core bi-layered scaffolds for engineering of vascularized osteon-like structures. Biomaterials 34, 8203–8212. 10.1016/j.biomaterials.2013.07.035 23896002

[B15] Chung R. Kalyon D. M. Yu X. Valdevit A. (2018). Segmental bone replacement via patient‐specific, three‐dimensional printed bioresorbable graft substitutes and their use as templates for the culture of mesenchymal stem cells under mechanical stimulation at various frequencies. Biotechnol. Bioeng. 115, 2365–2376. 10.1002/bit.26780 29940090

[B16] Cvetkovic V. J. Najman S. Rajkovic J. Zabar A. Vasiljevic P. Djordjevic L. (2013). A comparison of the microarchitecture of lower limb long bones between some animal models and humans: a review. Vet. Med. (Praha) 58, 339–351. 10.17221/6914-vetmed

[B17] Di Luca A. Ostrowska B. Lorenzo-Moldero I. Lepedda A. Swieszkowski W. Van Blitterswijk C. (2016a). Gradients in pore size enhance the osteogenic differentiation of human mesenchymal stromal cells in three-dimensional scaffolds. Sci. Rep. 6, 22898. 10.1038/srep22898 26961859 PMC4790631

[B18] Di Luca A. Longoni A. Criscenti G. Mota C. van Blitterswijk C. Moroni L. (2016b). Toward mimicking the bone structure: design of novel hierarchical scaffolds with a tailored radial porosity gradient. Biofabrication 8, 045007. 10.1088/1758-5090/8/4/045007 27725338

[B19] Dong X. N. Guo X. E. (2001). Predicting a power law between elastic modulus and porosity in cortical bone: a micromechanics model. Am. Soc. Mech. Eng. Bioeng. Div. BED 51, 65–66.

[B20] Ebrahimi H. Rabinovich M. Vuleta V. Zalcman D. Shah S. Dubov A. (2012). Biomechanical properties of an intact, injured, repaired, and healed femur: an experimental and computational study. J. Mech. Behav. Biomed. Mater. 16, 121–135. 10.1016/j.jmbbm.2012.09.005 23182385

[B21] Effanga V. E. Akilbekova D. Mukasheva F. Zhao X. Kalyon D. M. Erisken C. (2024). *In vitro* investigation of 3D printed hydrogel scaffolds with electrospun tidemark component for modeling osteochondral interface. Gels Basel, Switzerland 10, 745. 10.3390/gels10110745 39590101 PMC11593412

[B22] El-Gizawy A. S. Ma X. Pfeiffer F. Schiffbauer J. D. Selly T. (2023). Characterization of microarchitectures, stiffness and strength of human trabecular bone using micro-computed tomography (Micro-CT) scans. BioMed 3, 89–100. 10.3390/biomed3010007

[B23] Ellis S. L. Grassinger J. Jones A. Borg J. Camenisch T. Haylock D. (2011). The relationship between bone, hemopoietic stem cells, and vasculature. Blood 118, 1516–1524. 10.1182/blood-2010-08-303800 21673348

[B24] Engelke K. Graeff W. Meiss L. Hahn M. Delling G. (1993). High spatial resolution imaging of bone mineral using computed microtomography. Comparison with microradiography and undecalcified histologic sections. Invest. Radiol. 28, 341–349. 10.1097/00004424-199304000-00016 7683009

[B25] Ergun A. Yu X. Valdevit A. Ritter A. Kalyon D. M. (2011a). *In vitro* analysis and mechanical properties of twin screw extruded single-layered and coextruded multilayered poly(caprolactone) scaffolds seeded with human fetal osteoblasts for bone tissue engineering. J. Biomed. Mater. Res. A 99, 354–366. 10.1002/jbm.a.33190 22021183

[B26] Ergun A. Kalyon D. Valdevit A. Ritter A. (2011b). Compressive fatigue behavior of osteoblast seeded tissue constructs of poly(caprolactone) multilayered scaffolds for bone graft substitute applications. in Orthopaedic research society transactions, 1850.

[B27] Ergun A. Yu X. Valdevit A. Ritter A. Kalyon D. M. (2012a). Radially and axially graded multizonal bone graft substitutes targeting critical-sized bone defects from polycaprolactone/hydroxyapatite/tricalcium phosphate. Tissue Eng. Part A 18, 2426–2436. 10.1089/ten.TEA.2011.0625 22764839 PMC3501112

[B28] Ergun A. Chung R. Ward D. Valdevit A. Ritter A. Kalyon D. M. (2012b). Unitary bioresorbable cage/core bone graft substitutes for spinal arthrodesis coextruded from polycaprolactone biocomposites. Ann. Biomed. Eng. 40, 1073–1087. 10.1007/s10439-011-0484-1 22179683

[B29] Erisken C. Kalyon D. M. Wang H. (2008a). Functionally graded electrospun polycaprolactone and β-tricalcium phosphate nanocomposites for tissue engineering applications. Biomaterials 29, 4065–4073. 10.1016/j.biomaterials.2008.06.022 18649939

[B30] Erisken C. Kalyon D. M. Wang H. (2008b). A hybrid twin screw extrusion/electrospinning method to process nanoparticle-incorporated electrospun nanofibres. Nanotechnology 19, 165302. 10.1088/0957-4484/19/16/165302 21825641

[B31] Erisken C. Kalyon D. M. Wang H. (2010). Viscoelastic and biomechanical properties of osteochondral tissue constructs generated from graded polycaprolactone and beta-tricalcium phosphate composites. J. Biomech. Eng. 132, 091013. 10.1115/1.4001884 20815647

[B32] Erisken C. Kalyon D. M. Wang H. Ornek-Ballanco C. Xu J. (2011). Osteochondral tissue formation through adipose-derived stromal cell differentiation on biomimetic polycaprolactone nanofibrous scaffolds with graded insulin and beta-glycerophosphate concentrations. Tissue Eng. Part A 17, 1239–1252. 10.1089/ten.TEA.2009.0693 21189068

[B33] Feng G. Todoh M. (2021). Effect of microstructure and bone mineral density on mechanical properties of cortical bone. Proc. Mater. Mech. Conf. 2021, OS0409. 10.1299/jsmemm.2021.os0409

[B34] Fratzl P. Gupta H. S. Paschalis E. P. Roschger P. (2004). Structure and mechanical quality of the collagen–mineral nano-composite in bone. J. Mater. Chem. 14, 2115–2123. 10.1039/b402005g

[B35] Gabet Y. Kohavi D. Kohler T. Baras M. Müller R. Bab I. (2008). Trabecular bone gradient in rat long bone metaphyses: mathematical modeling and application to morphometric measurements and correction of implant positioning. J. Bone Min. Res. 23, 48–57. 10.1359/jbmr.070901 17892373

[B36] Gallab M. Le P. T. M. Shintani S. A. Takadama H. Ito M. Kitagaki H. (2024). Mechanical, bioactive, and long-lasting antibacterial properties of a Ti scaffold with gradient pores releasing iodine ions. Biomater. Adv. 158, 213781. 10.1016/j.bioadv.2024.213781 38335763

[B37] Gauthier R. Langer M. Follet H. Olivier C. Gouttenoire P. J. Helfen L. (2018). 3D micro structural analysis of human cortical bone in paired femoral diaphysis, femoral neck and radial diaphysis. J. Struct. Biol. 204, 182–190. 10.1016/j.jsb.2018.08.006 30107234

[B38] Harrison K. D. Hiebert B. D. Panahifar A. Andronowski J. M. Ashique A. M. King G. A. (2020). Cortical bone porosity in rabbit models of osteoporosis. J. Bone Min. Res. 35, 2211–2228. 10.1002/jbmr.4124 32614975 PMC7702175

[B39] Huiskes R. (1982). On the modelling of long bones in structural analyses. J. Biomech. 15, 65–69. 10.1016/0021-9290(82)90036-7 7061529

[B40] Huiskes H. W. J. Janssen J. D. Slooff T. J. J. H. (1983). A detailed comparison of experimental and theoretical stress-analysis of a human femur. in Mechanical properties of bone. New York: American Society of Mechanical Engineers, 211–234. Available online at: https://research.tue.nl/en/publications/a-detailed-comparison-of-experimental-and-theoretical-stress-anal (Accessed July 20, 2025).

[B41] Jin Y. Li J. Fan H. Du J. He Y. (2025). Biomechanics and mechanobiology of additively manufactured porous load-bearing bone implants. Small 21, e2409955. 10.1002/smll.202409955 40244634

[B42] Jindal M. (2018). Bone density versus bone quality as a predictor of bone strength. Orthop. Rheumatol. Open Access J. 12, 555830. 10.19080/oroaj.2018.12.555830

[B43] Johannesdottir F. Poole K. E. S. Reeve J. Siggeirsdottir K. Aspelund T. Mogensen B. (2011). Distribution of cortical bone in the femoral neck and hip fracture: a prospective case-control analysis of 143 incident hip fractures; the AGES-REYKJAVIK study. Bone 48, 1268–1276. 10.1016/j.bone.2011.03.776 21473947 PMC3129599

[B44] Kadyr S. Nurmanova U. Khumyrzakh B. Zhakypbekova A. Saginova D. Daniyeva N. (2024). Braided biomimetic PCL grafts for anterior cruciate ligament repair and regeneration. Biomed. Mater. 19. 10.1088/1748-605X/ad2555 38306680

[B45] Kadyr S. Khumyrzakh B. Naz S. Abdossova A. Askarbek B. Kalyon D. M. (2025). Hydrogels for osteochondral interface regeneration: biomaterial types, processes, and animal models. Gels 12, 24. 10.3390/gels12010024 41590050 PMC12841089

[B46] Karageorgiou V. Kaplan D. (2005). Porosity of 3D biomaterial scaffolds and osteogenesis. Biomaterials 26, 5474–5491. 10.1016/j.biomaterials.2005.02.002 15860204

[B47] Kaya S. Basta-Pljakic J. Seref-Ferlengez Z. Majeska R. J. Cardoso L. Bromage T. G. (2017). Lactation-induced changes in the volume of osteocyte lacunar-canalicular space alter mechanical properties in cortical bone tissue. J. Bone Min. Res. 32, 688–697. 10.1002/jbmr.3044 27859586 PMC5395324

[B48] Krishna B. V. Bose S. Bandyopadhyay A. (2007). Low stiffness porous Ti structures for load-bearing implants. Acta Biomater. 3, 997–1006. 10.1016/j.actbio.2007.03.008 17532277

[B49] Leong K. Chua C. Sudarmadji N. Yeong W. (2008). Engineering functionally graded tissue engineering scaffolds. J. Mech. Behav. Biomed. Mater. 1, 140–152. 10.1016/j.jmbbm.2007.11.002 19627779

[B50] Li Y. Chen S. K. Li L. Qin L. Wang X. L. Lai Y. X. (2015). Bone defect animal models for testing efficacy of bone substitute biomaterials. J. Orthop. Transl. 3, 95–104. 10.1016/j.jot.2015.05.002 30035046 PMC5982383

[B51] Li J. Xu T. Hou W. Liu F. Qing W. Huang L. (2020). The response of host blood vessels to graded distribution of macro-pores size in the process of ectopic osteogenesis. Mater. Sci. Eng. C 109, 110641. 10.1016/j.msec.2020.110641 32228974

[B52] Li Y. Han Q. Chen H. Yang W. Xu Y. Zhang Y. (2024). Advanced biomimetic design strategies for porous structures promoting bone integration with additive-manufactured Ti6Al4V scaffolds. J. Mater. Res. Technol. 32, 1901–1915. 10.1016/j.jmrt.2024.08.040

[B53] Lloyd A. A. Wang Z. X. Donnelly E. (2015). Multiscale contribution of bone tissue material property heterogeneity to trabecular bone mechanical behavior. J. Biomech. Eng. 137, 108011–108018. 10.1115/1.4029046 25383615 PMC4296240

[B54] Lorentzon M. (2020). The importance and possible clinical impact of measuring trabecular and cortical bone microstructure to improve fracture risk prediction. J. Bone Min. Res. 35, 831–832. 10.1002/jbmr.3940 31910297

[B55] Loundagin L. L. Pohl A. J. Edwards W. B. (2021). Stressed volume estimated by finite element analysis predicts the fatigue life of human cortical bone: the role of vascular canals as stress concentrators. Bone 143, 115647. 10.1016/j.bone.2020.115647 32956853

[B56] Lu H. H. Subramony S. D. Boushell M. K. Zhang X. (2010). Tissue engineering strategies for the regeneration of orthopedic interfaces. Ann. Biomed. Eng. 38, 2142–2154. 10.1007/s10439-010-0046-y 20422291 PMC3665605

[B57] Martin R. B. (1991). Determinants of the mechanical properties of bones. J. Biomech. 24 (Suppl. 1), 79–88. 10.1016/0021-9290(91)90379-2 1842337

[B58] Martin R. M. Correa P. H. S. (2010). Bone quality and osteoporosis therapy. Arq. Bras. Endocrinol. Metabol. 54, 186–199. 10.1590/s0004-27302010000200015 20485908

[B59] Mikos A. G. Herring S. W. Ochareon P. Elisseeff J. Lu H. H. Kandel R. (2006). Engineering complex tissues. Tissue Eng. 12, 3307–3339. 10.1089/ten.2006.12.3307 17518671 PMC2821210

[B60] Milovanovic P. Rakocevic Z. Djonic D. Zivkovic V. Hahn M. Nikolic S. (2014). Nano-structural, compositional and micro-architectural signs of cortical bone fragility at the superolateral femoral neck in elderly hip fracture patients vs. healthy aged controls. Exp. Gerontol. 55, 19–28. 10.1016/j.exger.2014.03.001 24614625

[B61] Nawathe S. Nguyen B. P. Barzanian N. Akhlaghpour H. Bouxsein M. L. Keaveny T. M. (2015). Cortical and trabecular load sharing in the human femoral neck. J. Biomech. 48, 816–822. 10.1016/j.jbiomech.2014.12.022 25582355

[B62] Nyman J. S. Gorochow L. E. Adam Horch R. Uppuganti S. Zein-Sabatto A. Manhard M. K. (2013). Partial removal of pore and loosely bound water by low-energy drying decreases cortical bone toughness in young and old donors. J. Mech. Behav. Biomed. Mater. 22, 136–145. 10.1016/j.jmbbm.2012.08.013 23631897 PMC3655090

[B63] Oshkour A. A. Osman N. A. A. Yau Y. H. Tarlochan F. Abas W. A. B. W. (2013). Design of new generation femoral prostheses using functionally graded materials: a finite element analysis. Proc. Inst. Mech. Eng. H. 227, 3–17. 10.1177/0954411912459421 23516951

[B64] Ozkan S. Kalyon D. M. Yu X. McKelvey C. A. Lowinger M. (2009). Multifunctional protein-encapsulated polycaprolactone scaffolds: fabrication and *in vitro* assessment for tissue engineering. Biomaterials 30, 4336–4347. 10.1016/j.biomaterials.2009.04.050 19481253

[B65] Ozkan S. Kalyon D. M. Yu X. (2010). Functionally graded β‐TCP/PCL nanocomposite scaffolds: *in vitro* evaluation with human fetal osteoblast cells for bone tissue engineering. J. Biomed. Mater. Res. Part A 92A, 1007–1018. 10.1002/jbm.a.32425 19296543

[B66] Palacio-Mancheno P. E. Larriera A. I. Doty S. B. Cardoso L. Fritton S. P. (2014). 3D assessment of cortical bone porosity and tissue mineral density using high-resolution µCT: effects of resolution and threshold method. J. Bone Min. Res. 29, 142–150. 10.1002/jbmr.2012 23775635 PMC3870034

[B67] Pazzaglia U. E. Congiu T. Sibilia V. Quacci D. (2014). Osteoblast-osteocyte transformation. A SEM densitometric analysis of endosteal apposition in rabbit femur. J. Anat. 224, 132–141. 10.1111/joa.12138 24251983 PMC3969057

[B68] Pearce R. H. Thompson J. P. Bebault G. M. Flak B. (1991). Magnetic resonance imaging reflects the chemical changes of aging degeneration in the human intervertebral disk. J. Rheumatol. Suppl. 27, 42–43. 2027127

[B69] Pearce A. Richards R. Milz S. Schneider E. Pearce S. (2007). Animal models for implant biomaterial research in bone: a review. Eur. Cells Mater. 13, 1–10. 10.22203/ecm.v013a01 17334975

[B70] Peyrin F. Muller C. Carillon Y. Nuzzo S. Bonnassie A. Briguet A. (2001). Synchrotron radiation µCT: a reference tool for the characterization of bone samples. Adv. Exp. Med. Biol. 496, 129–142. 10.1007/978-1-4615-0651-5_14 11783615

[B71] Pompe W. Worch H. Epple M. Friess W. Gelinsky M. Greil P. (2003). Functionally graded materials for biomedical applications. Mater. Sci. Eng. A 362, 40–60. 10.1016/s0921-5093(03)00580-x

[B72] Puppi D. Chiellini F. Piras A. M. Chiellini E. (2010). Polymeric materials for bone and cartilage repair. Prog. Polym. Sci. 35, 403–440. 10.1016/j.progpolymsci.2010.01.006

[B73] Rachidi M. Breban S. Benhamou C. L. (2008). Les enjeux de la microarchitecture osseuse. J. Soc. Biol. 202, 265–273. 10.1051/jbio:2008035 19094925

[B74] Renders G. A. P. Mulder L. van Ruijven L. J. van Eijden T. M. G. J. (2007). Porosity of human mandibular condylar bone. J. Anat. 210, 239–248. 10.1111/j.1469-7580.2007.00693.x 17331174 PMC2100285

[B75] Rizzoli R. (2010). Microarchitecture in focus. Osteoporos. Int. 21 (Suppl. 2), 403–406. 10.1007/s00198-010-1242-1 20464373

[B76] Scarano A. Khater A. G. A. Gehrke S. A. Inchingolo F. Tari S. R. (2024). Animal models for investigating osseointegration: an overview of implant research over the last three decades. J. Funct. Biomater. 15, 83. 10.3390/jfb15040083 38667540 PMC11051165

[B77] Schindeler A. Mills R. J. Bobyn J. D. Little D. G. (2018). Preclinical models for orthopedic research and bone tissue engineering. J. Orthop. Res. 36, 832–840. 10.1002/jor.23824 29205478

[B78] Schmidutz F. Milz S. Schiuma D. Richards R. G. Windolf M. Sprecher C. M. (2021). Cortical parameters predict bone strength at the tibial diaphysis, but are underestimated by HR-pQCT and μCT compared to histomorphometry. J. Anat. 238, 669–678. 10.1111/joa.13337 33084063 PMC7855080

[B79] Schneider P. Voide R. Stampanoni M. Donahue L. R. Müller R. (2013). The importance of the intracortical canal network for murine bone mechanics. Bone 53, 120–128. 10.1016/j.bone.2012.11.024 23219945

[B80] Seeman E. (2017). Overview of bone microstructure, and treatment of bone fragility in chronic kidney disease. Nephrology 22, 34–35. 10.1111/nep.13024 28429552

[B81] Seidi A. Ramalingam M. Elloumi-Hannachi I. Ostrovidov S. Khademhosseini A. (2011). Gradient biomaterials for soft-to-hard interface tissue engineering. Acta Biomater. 7, 1441–1451. 10.1016/j.actbio.2011.01.011 21232635

[B82] Seitzhaparova B. Timur L. Mirasbek B. Kadyr S. Lesbekov T. Zhakypbekova A. (2025). Rabbit heart bioartificial tissue: perfusion decellularization and characterization. Biomed. Phys. Eng. Express 11, 015033. 10.1088/2057-1976/ad99de 39626314

[B83] Senturk-Ozer S. Aktas S. He J. Fisher F. T. Kalyon D. M. (2016). Nanoporous nanocomposite membranes via hybrid twin-screw extrusion—multijet electrospinning. Nanotechnology 28, 025301. 10.1088/0957-4484/28/2/025301 27905320

[B84] Smatov S. Mukasheva F. Erisken C. (2023). Collagen fibril diameter distribution of sheep anterior cruciate ligament. Polym. (Basel) 15, 752. 10.3390/polym15030752 36772054 PMC9920528

[B85] Someya K. Mochizuki T. Katsumi R. Hokari S. Tanifuji O. Kobayashi K. (2020). Correlation between cortical thickness and bowing of the femoral diaphysis in healthy elderly people. Osteoarthr. Cartil. 28, S216–S217. 10.1016/j.joca.2020.02.353

[B86] Sumner D. R. Turner T. M. Igloria R. Urban R. M. Galante J. O. (1998). Functional adaptation and ingrowth of bone vary as a function of hip implant stiffness. J. Biomech. 31, 909–917. 10.1016/s0021-9290(98)00096-7 9840756

[B87] Surowiec R. K. Allen M. R. Wallace J. M. (2022). Bone hydration: how we can evaluate it, what can it tell us, and is it an effective therapeutic target? Bone Rep. 16, 101161. 10.1016/j.bonr.2021.101161 35005101 PMC8718737

[B88] Tourlomousis F. Ding H. Kalyon D. M. Chang R. C. (2017). Melt electrospinning writing process guided by a “Printability Number”. J. Manuf. Sci. Eng. 139, 081004. 10.1115/1.4036348

[B89] Tourlomousis F. Jia C. Karydis T. Mershin A. Wang H. Kalyon D. M. (2019). Machine learning metrology of cell confinement in melt electrowritten three-dimensional biomaterial substrates. Microsystems Nanoeng. 5, 15. 10.1038/s41378-019-0055-4 31057942 PMC6431680

[B90] Uhthoff H. K. Poitras P. Backman D. S. (2006). Internal plate fixation of fractures: short history and recent developments. J. Orthop. Sci. 11, 118–126. 10.1007/s00776-005-0984-7 16568382 PMC2780616

[B91] Uniyal P. Sihota P. Tikoo K. Kumar N. (2021). Anatomical variation in intracortical canal network microarchitecture and its influence on bone fracture risk. J. Mech. Behav. Biomed. Mater. 123, 104770. 10.1016/j.jmbbm.2021.104770 34392038

[B92] Velioglu Z. B. Pulat D. Demirbakan B. Ozcan B. Bayrak E. Erisken C. (2019). 3D-printed poly(lactic acid) scaffolds for trabecular bone repair and regeneration: scaffold and native bone characterization. Connect. Tissue Res. 60, 274–282. 10.1080/03008207.2018.1499732 30058375

[B93] Voide R. van Lenthe G. H. Schneider P. Thurner P. J. Wyss P. Sennhauser U. (2006). Functional microimaging: an integrated approach for advanced bone biomechanics and failure analysis. in Medical Imaging 2006: Physiology, Function, and Structure from Medical Images 6143, 10.1117/12.650485

[B94] Wancket L. M. (2015). Animal models for evaluation of bone implants and devices. Vet. Pathol. 52, 842–850. 10.1177/0300985815593124 26163303

[B95] Wang S. Zhou X. Liu L. Shi Z. Hao Y. (2020). On the design and properties of porous femoral stems with adjustable stiffness gradient. Med. Eng. Phys. 81, 30–38. 10.1016/j.medengphy.2020.05.003 32505662

[B96] Wegrzyn J. Roux J. P. Arlot M. E. Boutroy S. Vilayphiou N. Guyen O. (2010). Role of trabecular microarchitecture and its heterogeneity parameters in the mechanical behavior of *ex vivo* human L3 vertebrae. J. Bone Min. Res. 25, 2324–2331. 10.1002/jbmr.164 20564249 PMC3179283

[B97] Yildirim N. Amanzhanova A. Kulzhanova G. Mukasheva F. Erisken C. (2023). Osteochondral interface: regenerative engineering and challenges. ACS Biomater. Sci. Eng. 9, 1205–1223. 10.1021/acsbiomaterials.2c01321 36752057

[B98] Zhang X. Xu S. Shen L. Li G. (2020). Factors affecting thermal stability of collagen from the aspects of extraction, processing and modification. J. Leather Sci. Eng. 2, 19. 10.1186/s42825-020-00033-0

[B99] Zhao X. Wang W. Yu X. Kalyon D. M. Erisken C. (2025). Advanced bioprinting of hydrogels with controlled mineral gradients for osteochondral interface. Tissue Eng. Part A 31, e1327–e1442.

[B100] Zhao X. Wang W. Yu X. Kalyon D. M. Erisken C. (2026). Bioextrusion of hydrogels with controlled mineral gradients for regenerative engineering of osteochondral interfaces. Bio-Design Manuf. 9, 122–136. 10.1631/bdm.2500291

[B101] Zimmermann E. A. Ritchie R. O. (2015). Bone as a structural material. Adv. Healthc. Mater. 4, 1287–1304. 10.1002/adhm.201500070 25865873

